# Supplementary material for: Human Mentalizing as Rational Probabilistic Inference
Source: Comput Brain Behav. 2025 Apr 9;8(4):517–34. doi: 10.1007/s42113-025-00244-w (PMC13298678; doi:10.1007/s42113-025-00244-w)
Supplement: Supplementary file 1 — (pdf 7916 KB) [file 42113_2025_244_MOESM1_ESM.pdf]

Supplementary Material for Human mentalizing as rational probabilistic inference

1 Computational Model

The information flow between the components of our model is illustrated in Fig. 1. Observations of an actor's behavior, combined with prior knowledge, are utilized to infer relevant mental states. These mental states —- including preferences and costs – are parameterized for a computationally rational agent, which adapts its policy to the task and returns simulated behavior. The BOLFI inference method interacts with the forward simulator ("World Simulator" in Fig. 1) to form a posterior distribution. Samples from this posterior distribution then parameterize a new world simulator, generating predictions of behavior in a novel (testing) environment, thereby accounting for the uncertainty captured during the inference process.

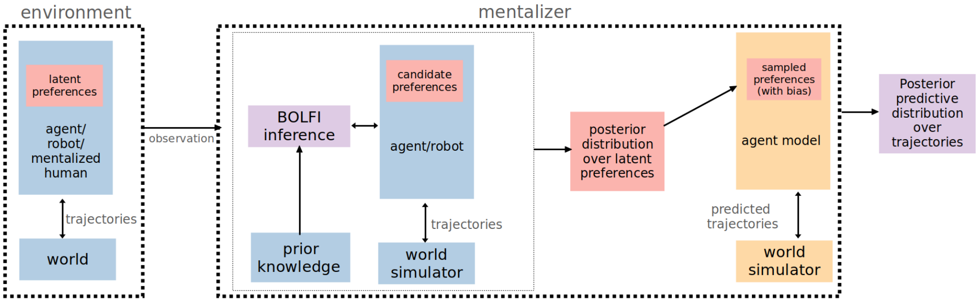

Fig. 1: The menalizer conducts the inference and probabilistic prediction process. The forward simulator assumes a computationally rational agent, meaning that given its preferences and environmental bounds, it generates an optimally adapted behavior trajectory. The mentalizer observes a trajectory, and uses Bayesian likelihood-free inference to incorporate prior knowledge, observations, and an internal simulation of the observed task behavior, producing a posterior distribution over latent mental states of the agent. The posterior is then sampled in a new simulated environment, resulting in a posterior predictive distribution over plausible trajectories that the agent might take in that new environment.

In the world simulator, the environment is represented as MDP process. The MDP representation of the environment describes how the state transfers to a new state when an action is conducted. It is represented as a triple $< \mathcal{S}, \mathcal{A}, \mathcal{R}, \mathcal{T} >$, including a state space $\mathcal{S}$, action space $\mathcal{A}$, transition probabilities $\mathcal{T}$, and a reward function $\mathcal{R}$. A state $s \in \mathcal{S}$ encodes the current position of the agent, the positions and status of the berries, and the color of the cells. The agent selects an action $a \in \mathcal{A}$ to transition to a new state $s' \in \mathcal{S}$ according to the transition probabilities $\mathcal{T}(s, a, s') = P(s'|s, a)$. The environment is deterministic: the agent's movement are determined by its intentions, and moving over a berry causes it to be collected. As a response to the agent's action, the reward function $\mathcal{R}(s, a) = r$ generates a numerical value $r \in \mathcal{R}$, given the state s and action a.

RL generates the human-like behavior in the MDP environment via Q-learning agent. The Bellman equation defines the optimal problem, and the Q learning agent solves the optimal problem of behavior via Temporal-Difference(TD) method. The update equation of value function is written as

$$V(s) \leftarrow V(s) + \alpha(\mathbf{R}(s, a) + \gamma V(s') - V(s)),$$

where step size $\alpha = 1$ and discount factor $\gamma = 0.99$ in our model. Exploring in the environment, the agent continuously updates the value of states, and forms a policy that acts to transfer to the state with highest value among all possible next states. The policy utilizes softmax policy on Q value $Q(s, a)$, which evaluates action a at state s. Q values are updated using the equation:

$$Q(s, a) \leftarrow Q(s, a) + \alpha(\mathbf{R}(s, a) + \gamma max_{a'} Q(s', a') - Q(s, a)).$$

At state s, the policy $\pi(a|s)$ calculates the probability of choosing action $P(a|s)$ via the softmax Q value of all the actions

$$\pi(a|s) = P(a|s) = softmax(Q(s, a)) = \frac{\exp(Q(s, a))}{\sum_{a' \in \mathcal{A}} \exp(Q(s, a'))}.$$

The value function $V(s)$ is related to the action-value function $Q(s, a)$ by

$$V(s) = \sum_{a} \pi(a|s) Q(s, a).$$

The inference utilizes Approximate Bayesian Computes (ABC) that samples to detect the posterior distribution. The samples are sent to the world simulator to generate trajectories, which are summarized and compared with the summarized observation. The summary statistics include the number of the gray cells and the white cells, whether the red berry is collected and whether the yellow berry is collected. We use euclidean distance to evaluate the discrepancy between summary statistics. No U-Turn Sampler (NUTS), a Hamiltonian Monte Carlo Method, is utilized in our model for inference. The hyperparameters required by BOLFI are the *initial_evidence* which

defines how many samples before inference, the *update_interval* which defines the frequency of updating hyperparameters and *bounds* that defines the range of the inferred parameters. The values of the parameters in our work are $initial\_evidence = 40$, $update\_interval = 20$ and *bounds* as $(-2, 0)$ for cost and $(0, 2)$ for preference. After inference, we sample 1000 points from posterior distribution for further prediction. As inference of each task is run for 10 times, we get 10000 samples for prediction. In prediction phases, all samples are sent to the corresponding test environments to produce prediction trajectories.

2 Experiment

2.1 Experiment Two

In experiment two, we demonstrate that humans are able to make probabilistic prediction of future events, based on observing the behavior of others and inferring their mental states. In total, experiment two contains 12 tasks, each including a stimulus and a test. The stimuli that include cues of robots' preference and cost and tests that indicate the probabilities of each trajectory are shown in Figure 2.

To better explain human behavior, we introduce a parameter of bias, which stands for the preference of berries over the dislikes of gray area. The parameter is represented as w_r in the model and previous section, short for weight of rewards. It is impacted by task environment, as illustrated in Experiment section. The value of w_r is shown in Figure 3 and the results are shown in Figure 4.

2.2 Experiment Three

In experiment three, we investigate the impact of knowledge accumulation, as well as uncertainty accumulation, on probabilistic prediction. By adding one more stimulus for each task, participants made their prediction based on multiple source of information. We have 30 tasks in total, of which the stimuli and test are shown in Figure 5. The value of w_r is shown in Figure 6, and the results of all tasks are shown in Figure 7.

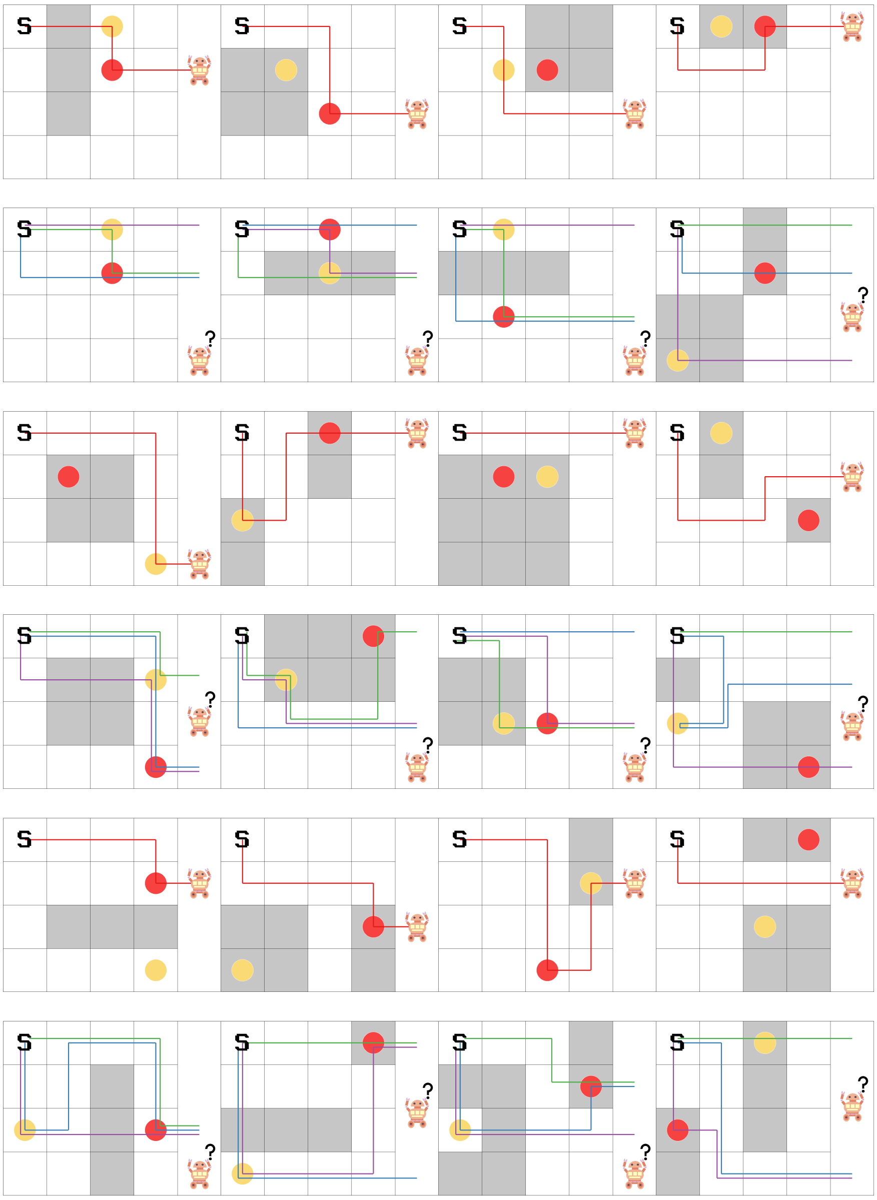

Fig. 2: Stimuli and tests of 12 tasks in experiment two.

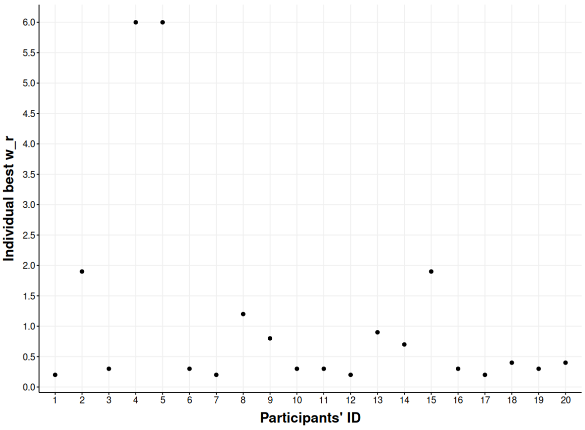

Fig. 3: The value of w_r in 12 tasks.

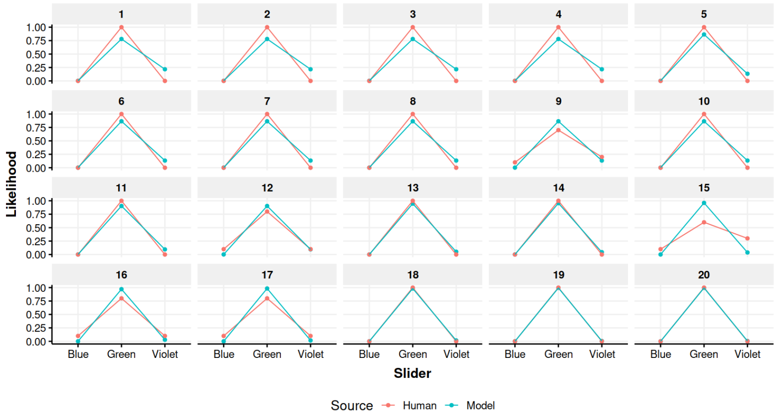

(a) Task 1

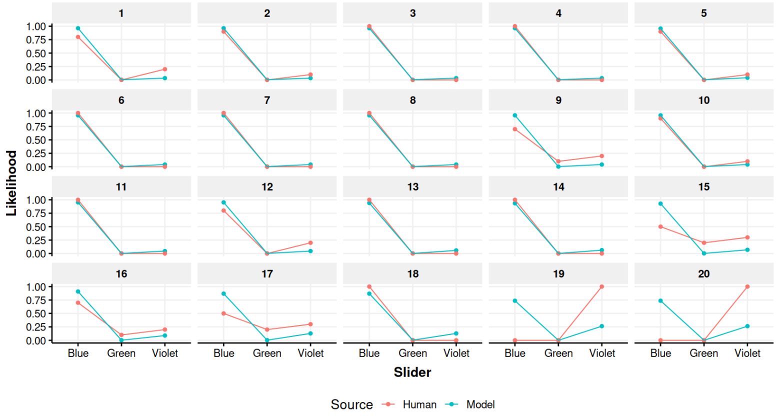

(b) Task 2

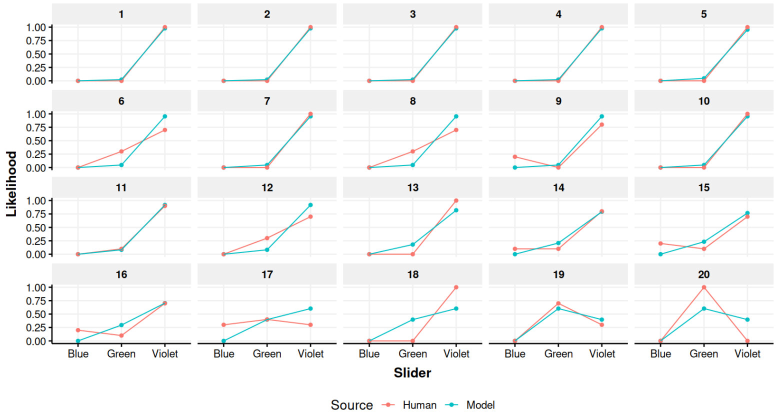

(c) Task 3

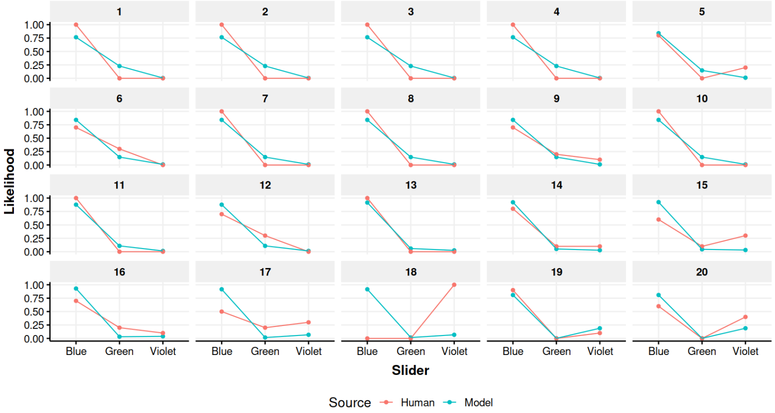

(d) Task 4

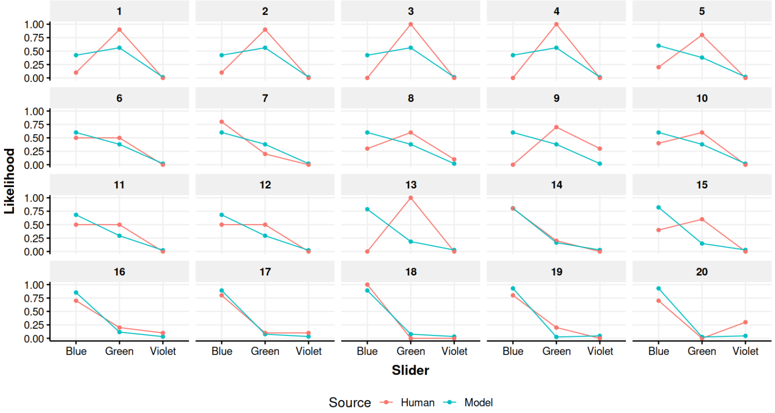

(e) Task 5

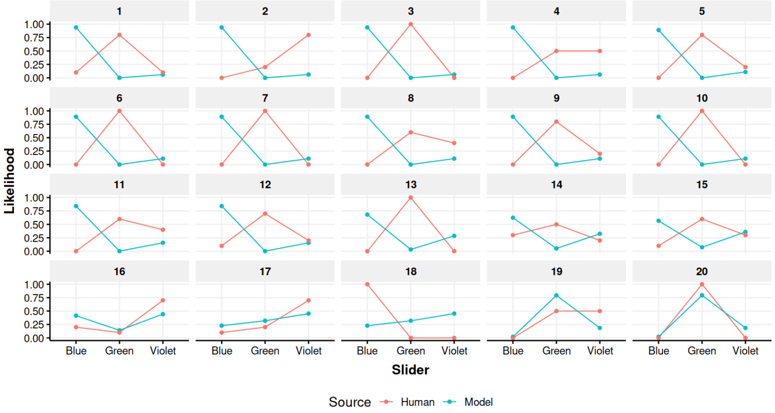

(f) Task 6

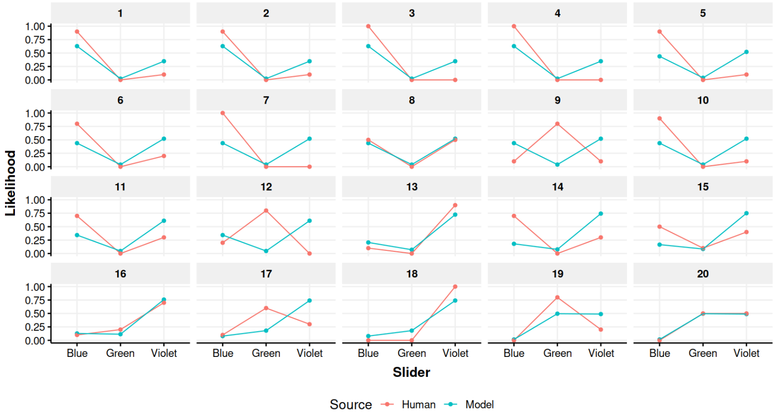

(g) Task 7

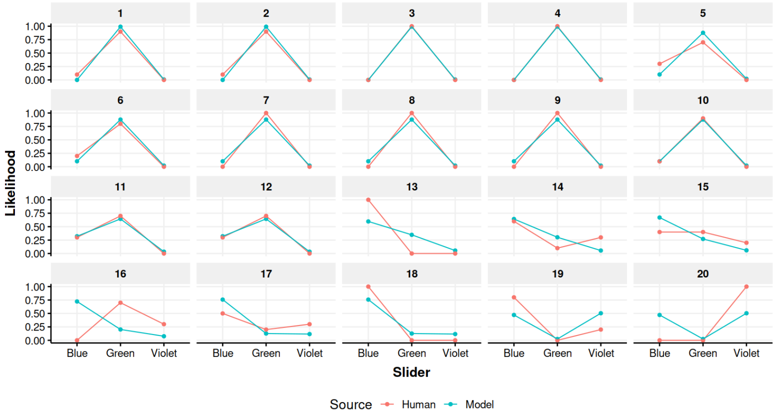

(h) Task 8

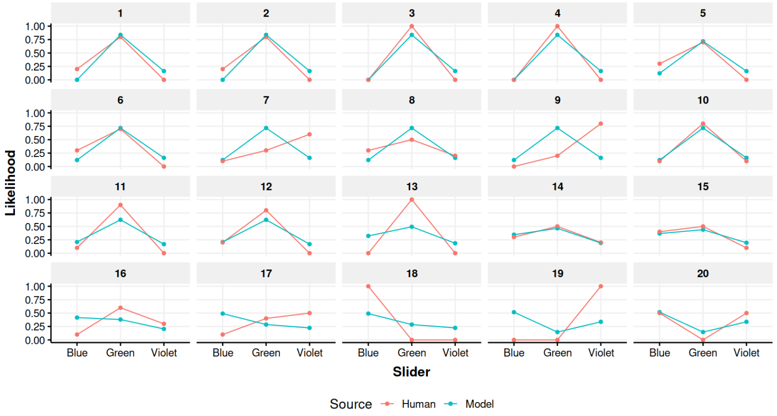

(i) Task 9

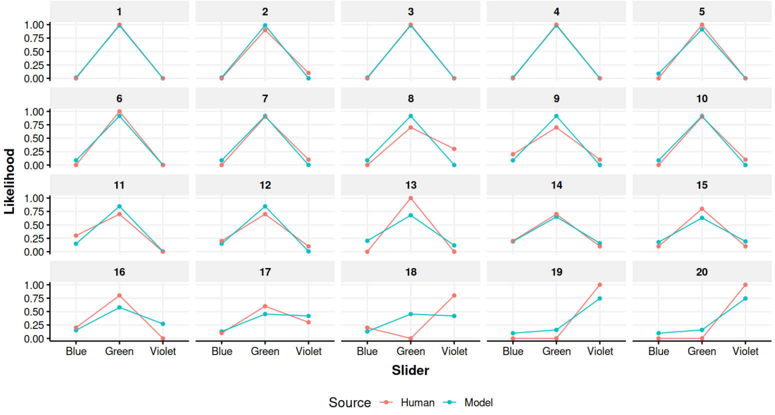

(j) Task 10

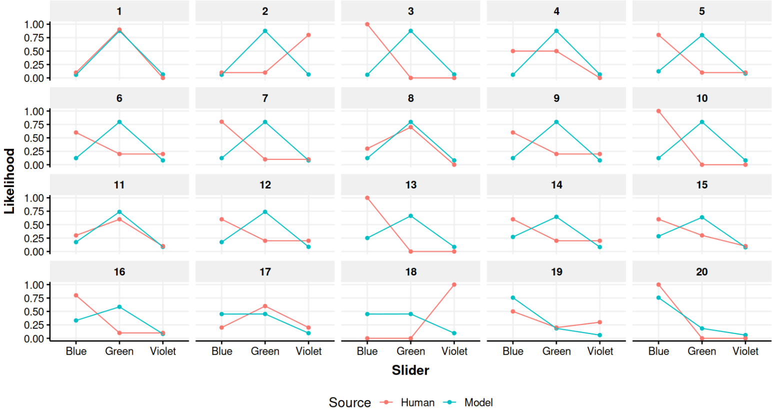

(k) Task 11

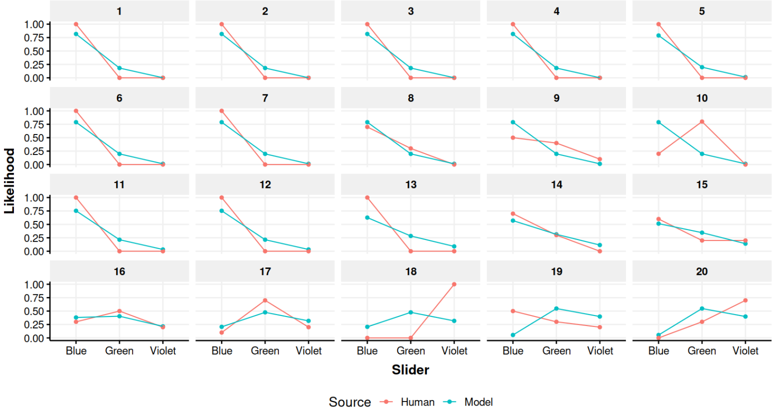

(l) Task 12

Fig. 4: Experiment Two Results

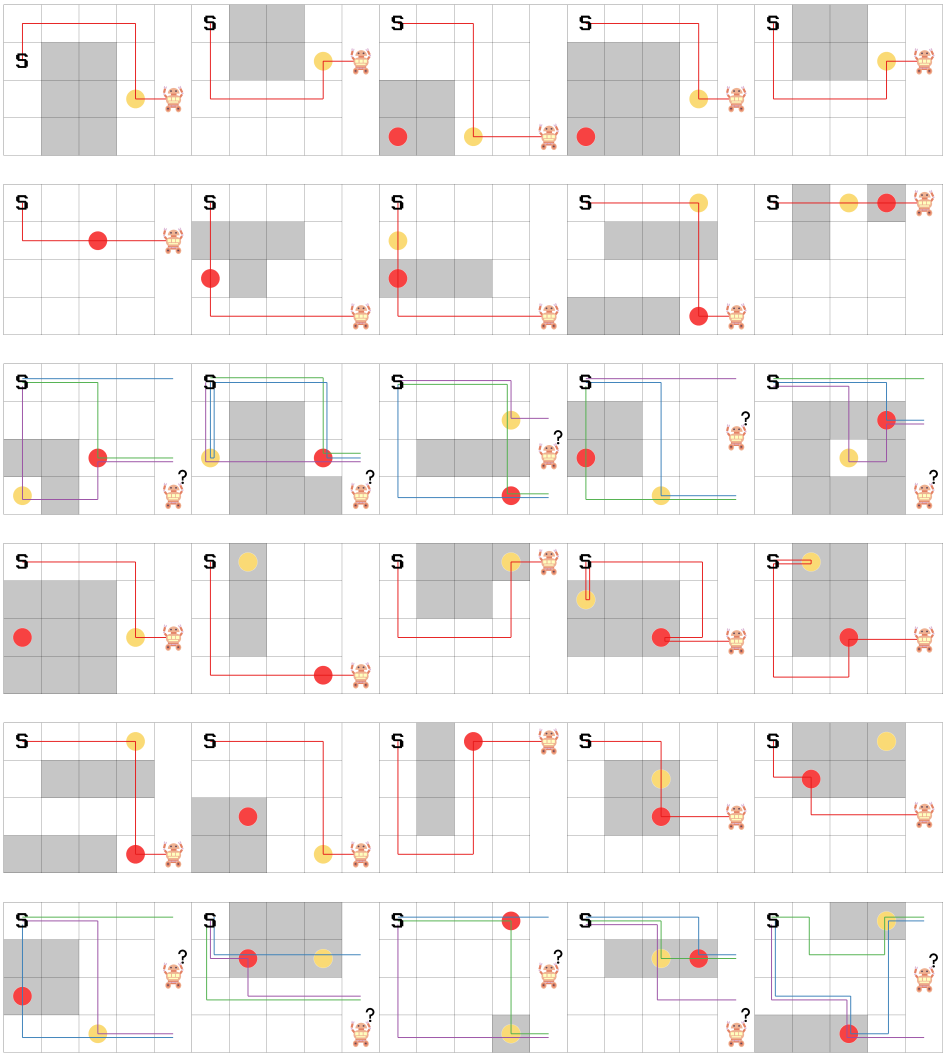

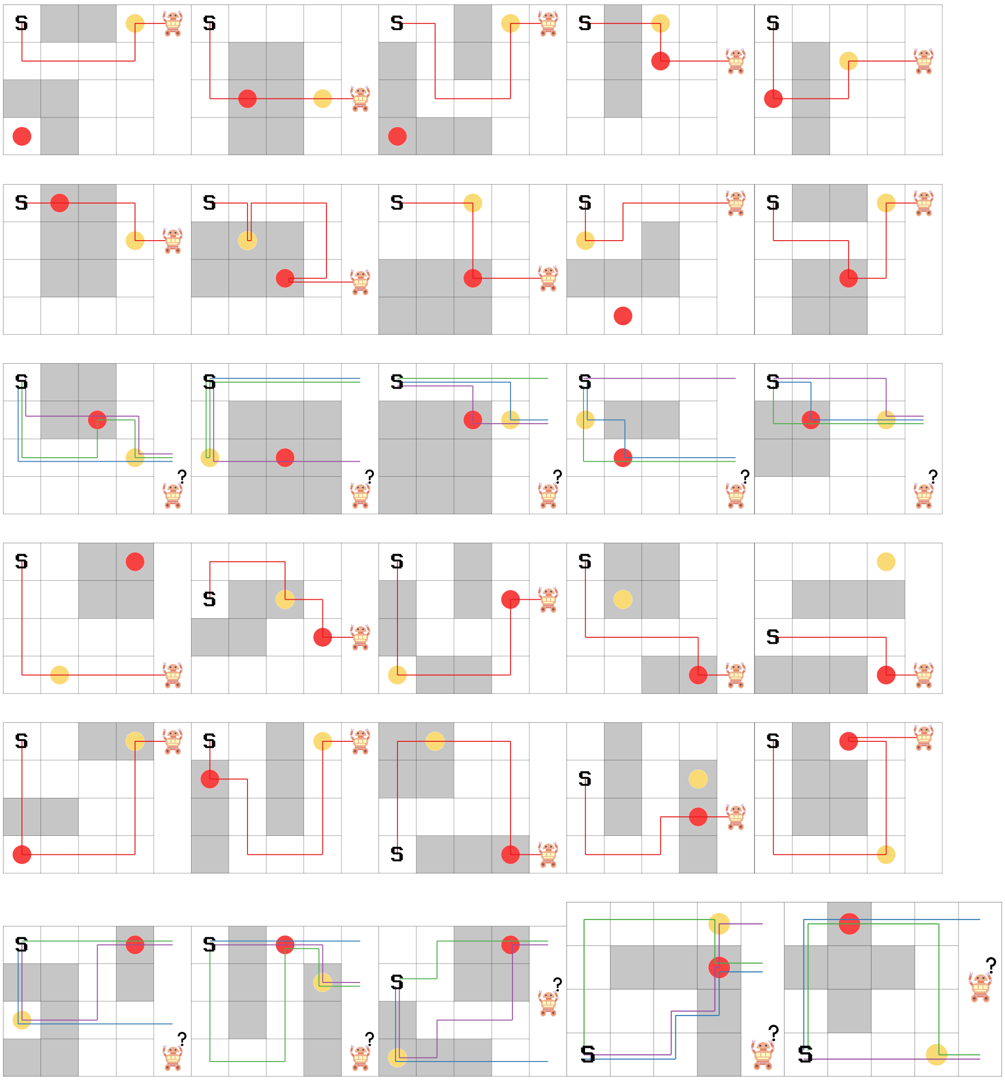

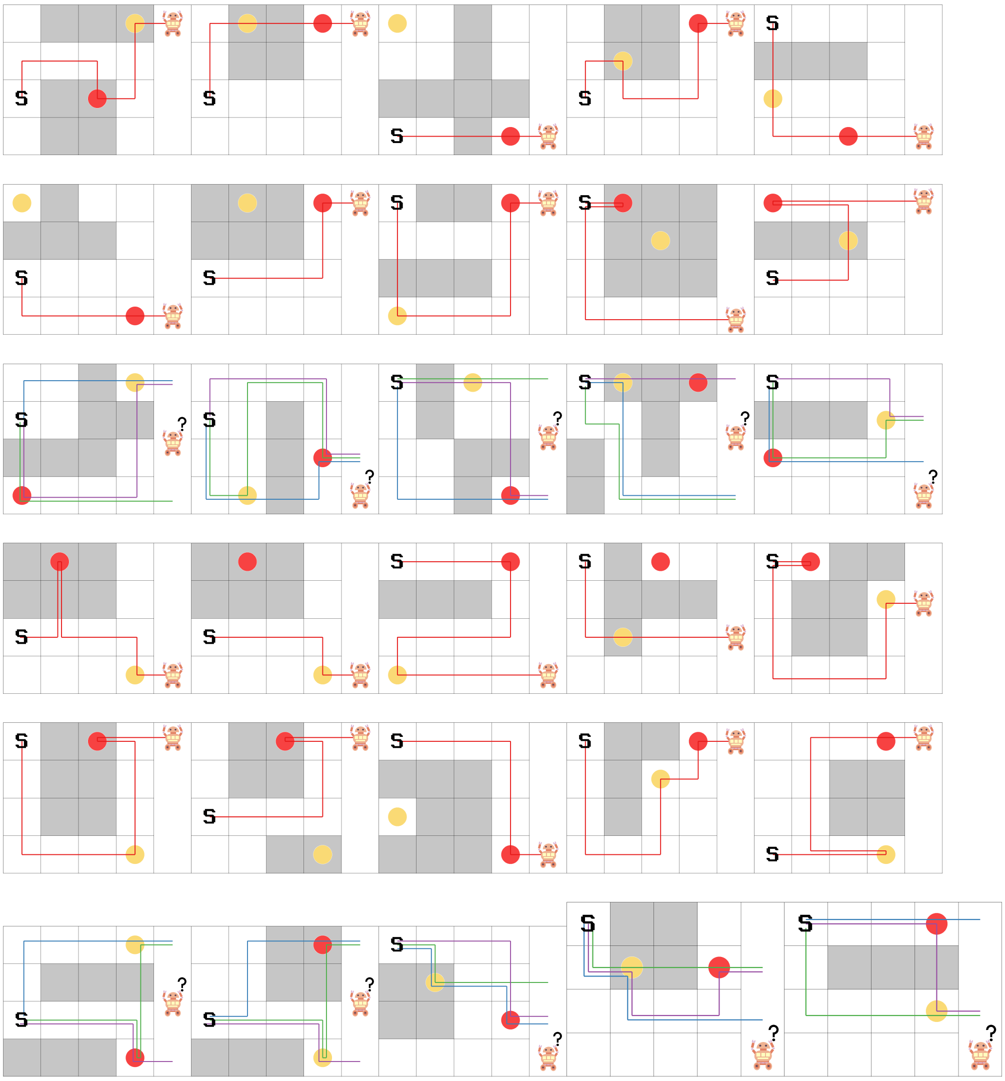

Fig. 5: Stimuli and tests of 30 tasks in experiment three.

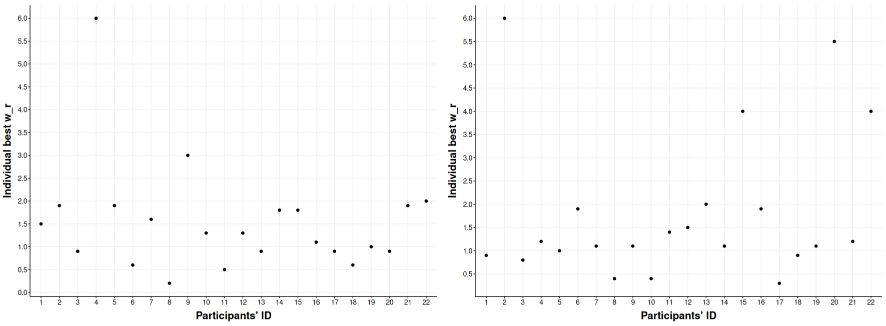

(a) Best individual w_r in Simultaneous Group (b) Best individual w_r in Sequential Group

Fig. 6: The value of w_r in 30 tasks.

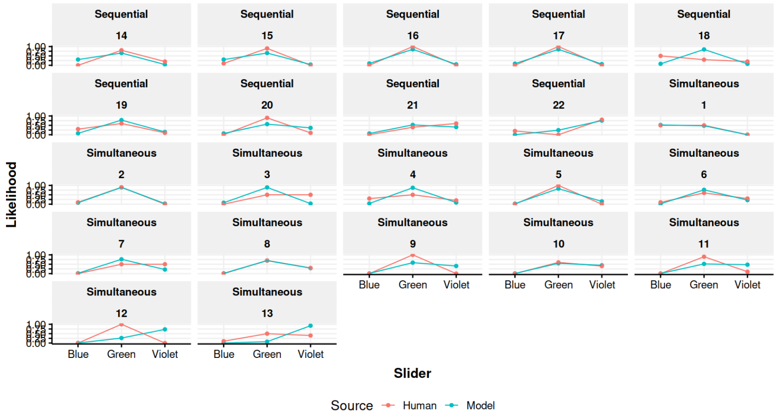

Task 1

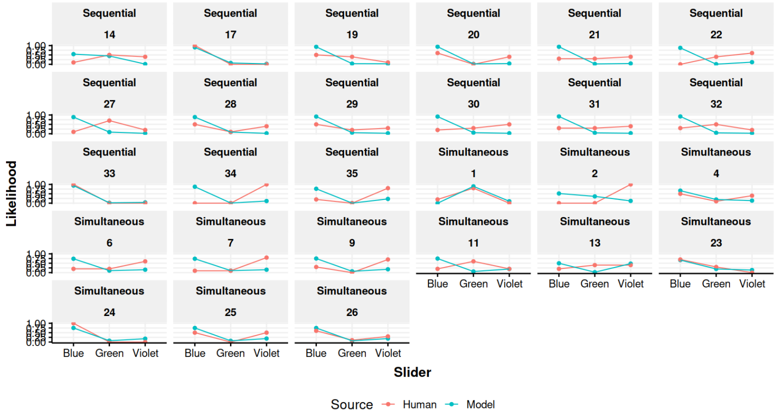

Task 2

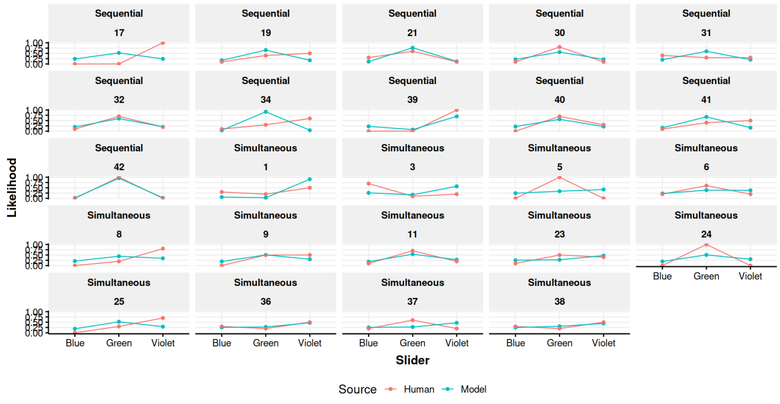

Task 3

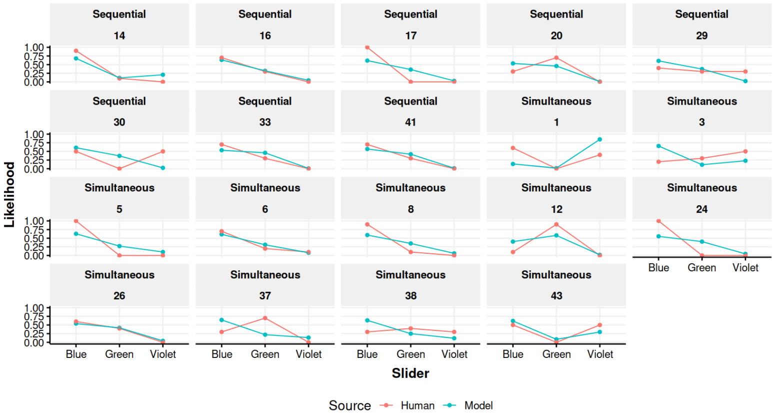

Task 4

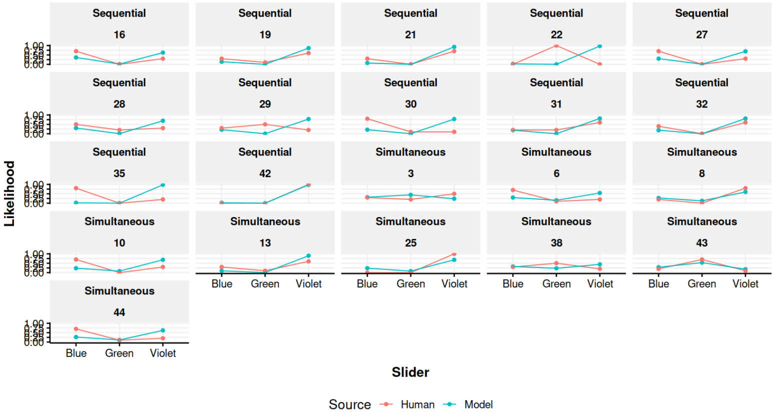

Task 5

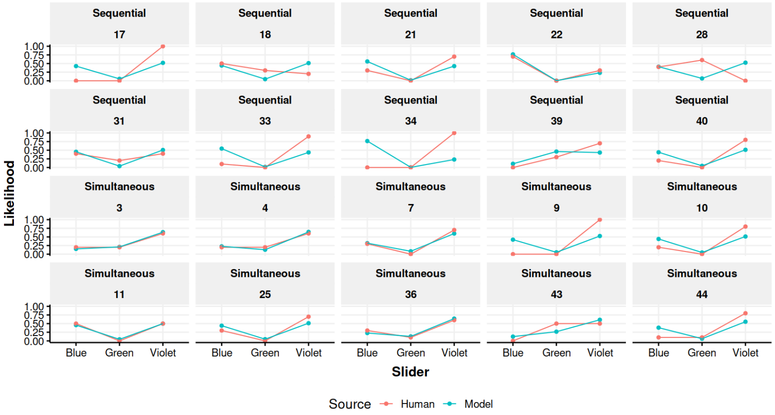

Task 6

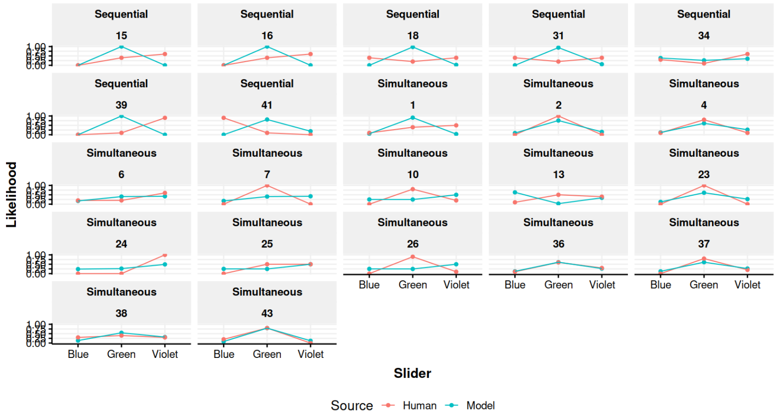

Task 7

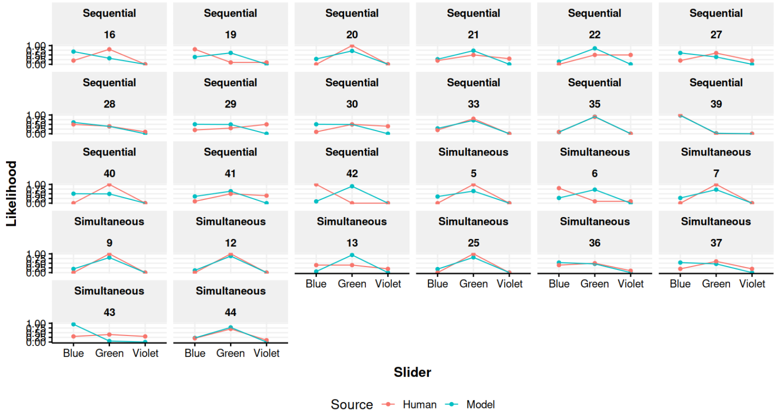

Task 8

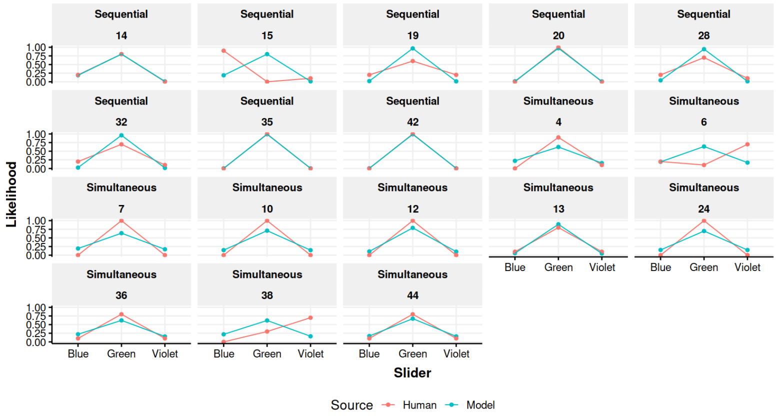

Task 9

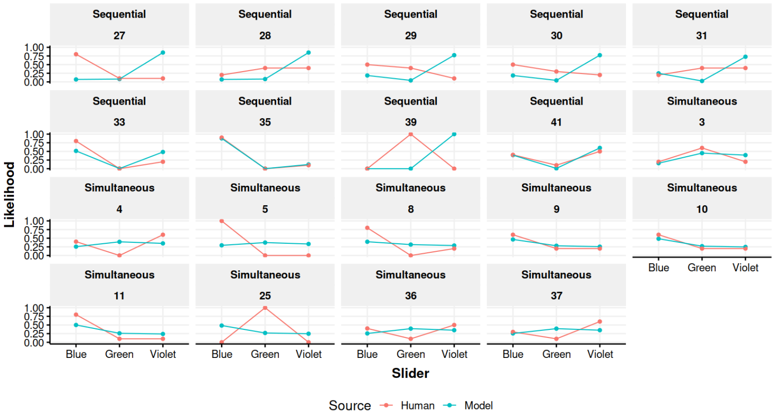

Task 10

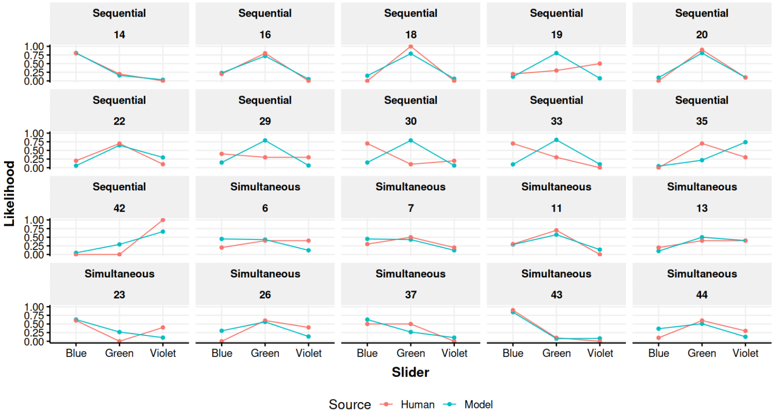

Task 11

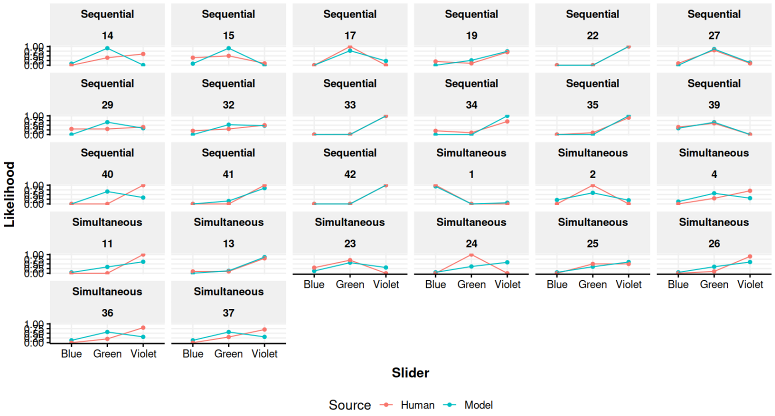

Task 12

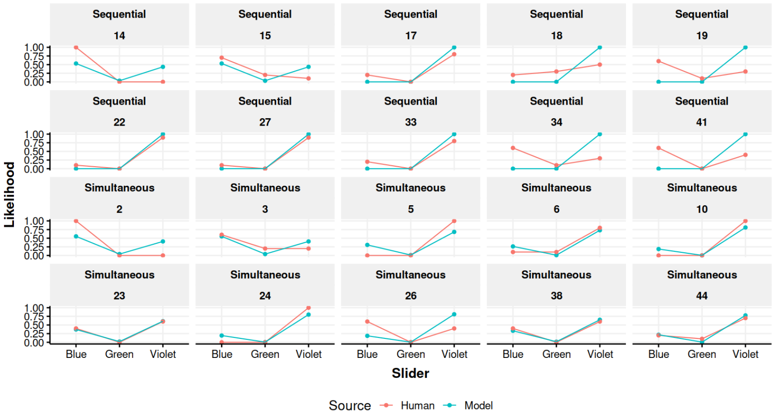

Task 13

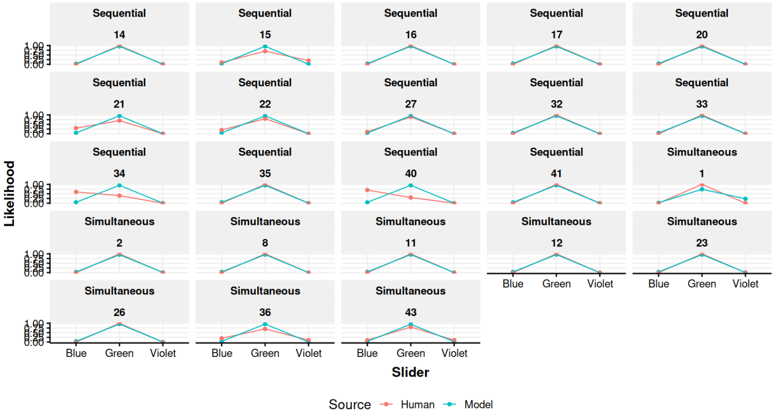

Task 14

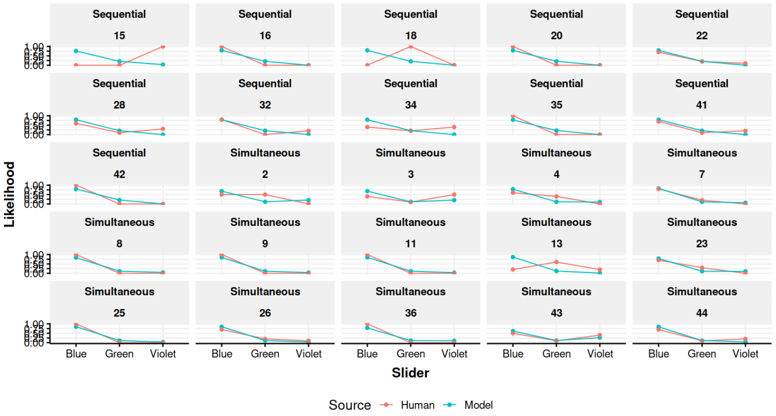

Task 15

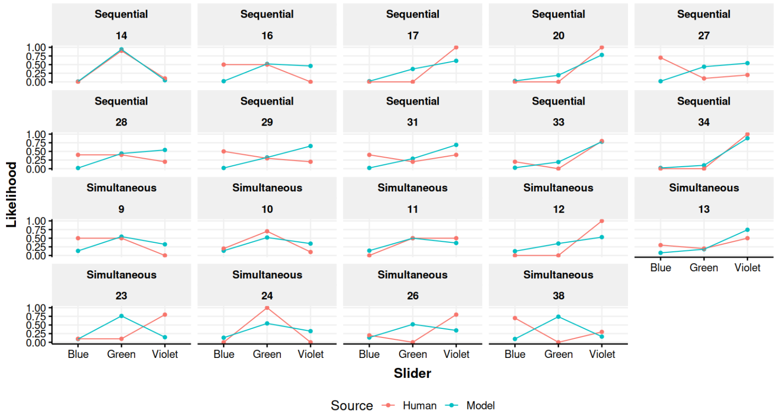

Task 16

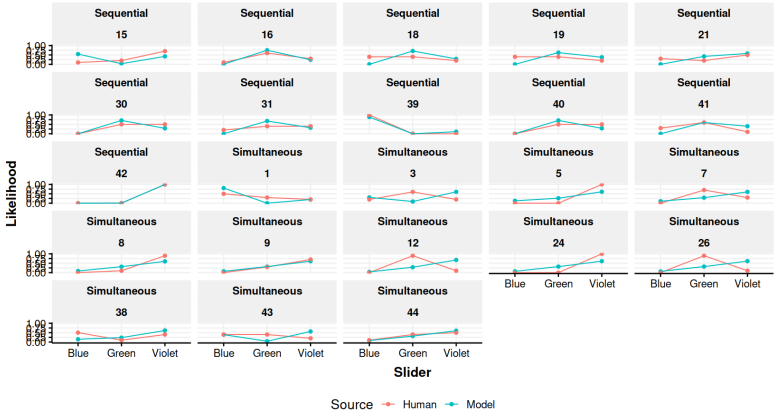

Task 17

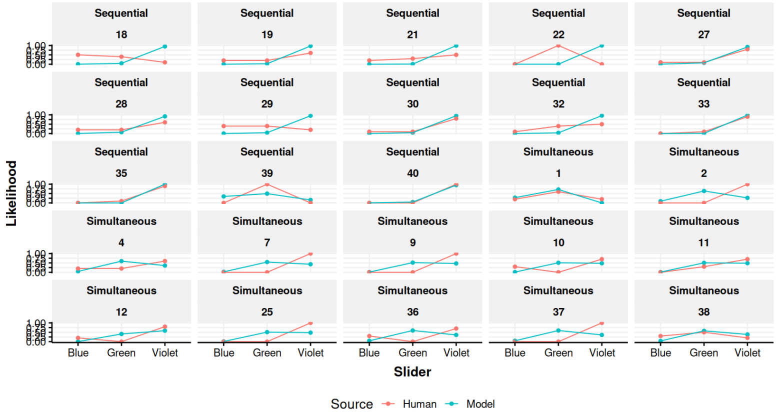

Task 18

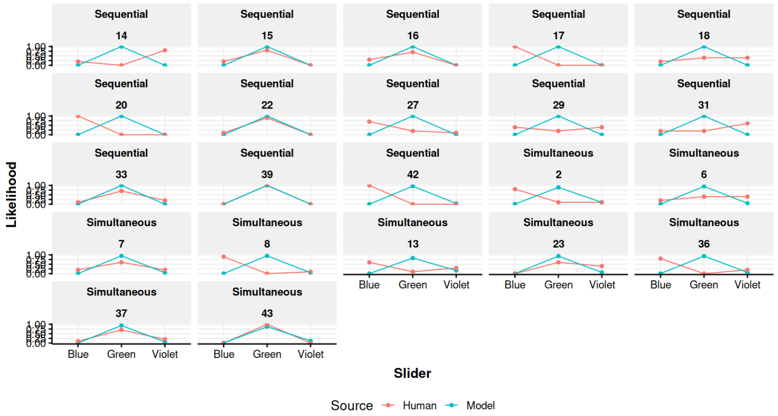

Task 19

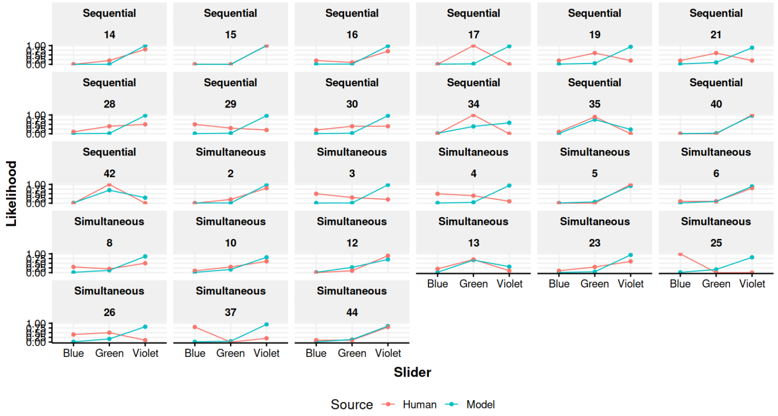

Task 20

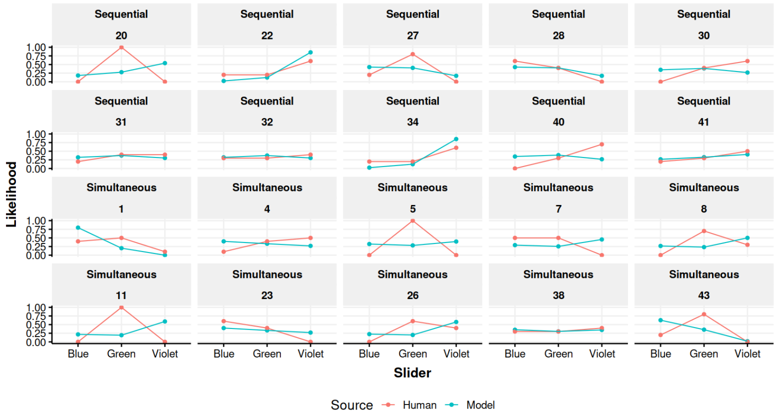

Task 21

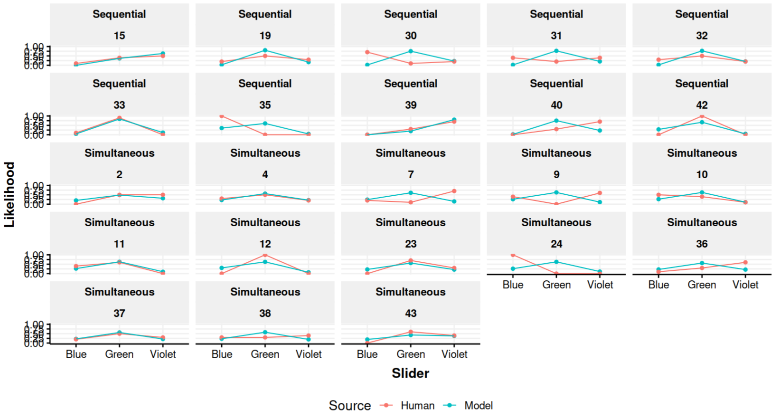

Task 22

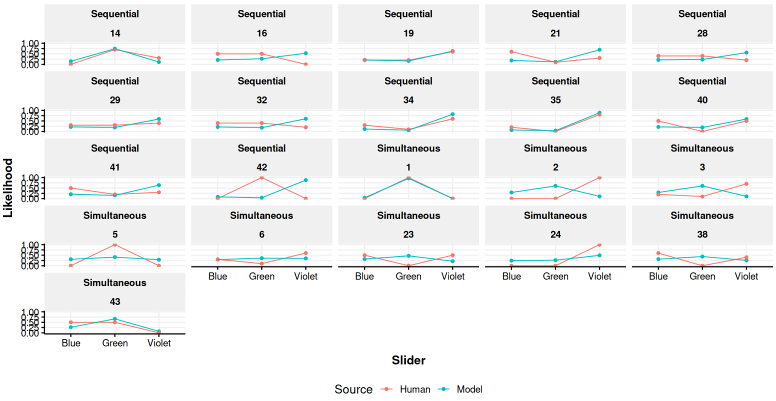

Task 23

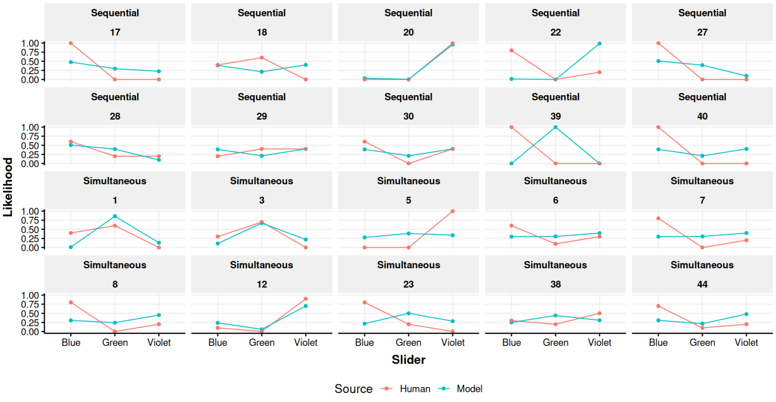

Task 24

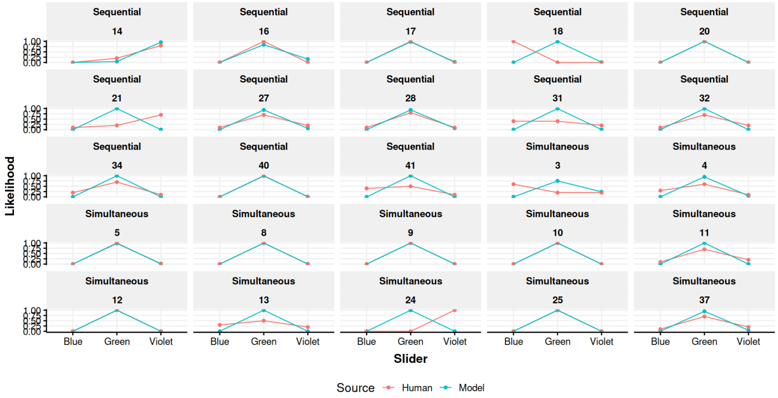

Task 25

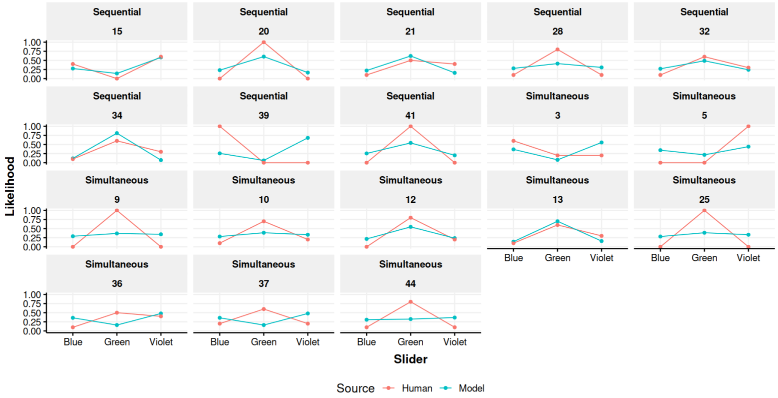

Task 26

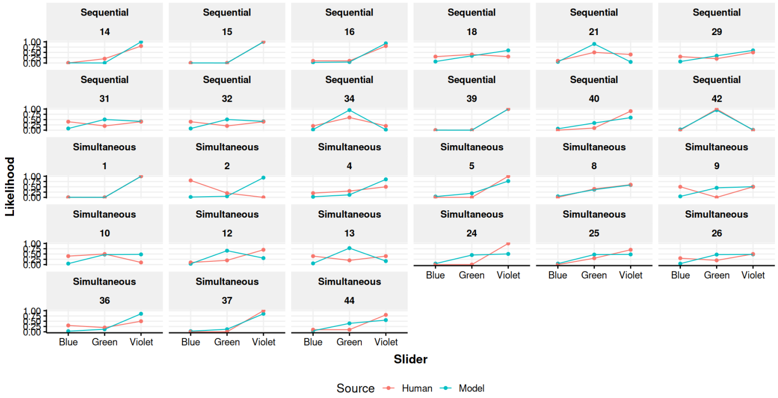

Task 27

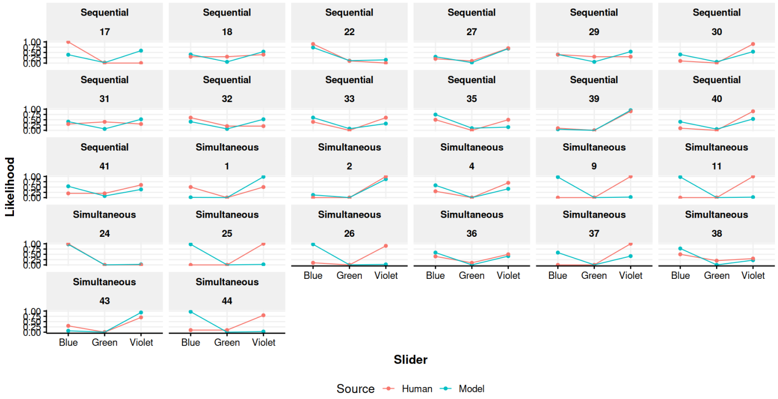

Task 28

Fig. 7: Experiment Three Results
